# Marine Polysaccharides as a Versatile Biomass for the Construction of Nano Drug Delivery Systems

**DOI:** 10.3390/md19060345

**Published:** 2021-06-16

**Authors:** Ying Sun, Xiaoli Ma, Hao Hu

**Affiliations:** 1 Institute of Biomedical Materials and Engineering, College of Materials Science and Engineering, Qingdao University, Qingdao 266071, China; sunying150996@163.com; 2 Qingdao Institute of Measurement Technology, Qingdao 266000, China; maxiaoli1989@yeah.net

**Keywords:** marine polysaccharide, drug delivery system, nanocarrier, cancer therapy

## Abstract

Marine biomass is a treasure trove of materials. Marine polysaccharides have the characteristics of biocompatibility, biodegradability, non-toxicity, low cost, and abundance. An enormous variety of polysaccharides can be extracted from marine organisms such as algae, crustaceans, and microorganisms. The most studied marine polysaccharides include chitin, chitosan, alginates, hyaluronic acid, fucoidan, carrageenan, agarose, and Ulva. Marine polysaccharides have a wide range of applications in the field of biomedical materials, such as drug delivery, tissue engineering, wound dressings, and sensors. The drug delivery system (DDS) can comprehensively control the distribution of drugs in the organism in space, time, and dosage, thereby increasing the utilization efficiency of drugs, reducing costs, and reducing toxic side effects. The nano-drug delivery system (NDDS), due to its small size, can function at the subcellular level in vivo. The marine polysaccharide-based DDS combines the advantages of polysaccharide materials and nanotechnology, and is suitable as a carrier for different pharmaceutical preparations. This review summarizes the advantages and drawbacks of using marine polysaccharides to construct the NDDS and describes the preparation methods and modification strategies of marine polysaccharide-based nanocarriers.

## 1. Introduction

Because the human body has a complex physiological environment and defense capabilities, whether it is administered by oral, intramuscular, or intravenous injection, the utilization of drugs has been severely weakened [1]. The hydrophilicity and hydrophobicity of drug molecules determine the absorption, distribution, metabolism, and excretion of drugs in the body [2]. The hydrophobic structure in some drug molecules, such as benzene rings, can increase the hydrophobicity of the drug. The development of nano-drug delivery systems (NDDSs) brings hope to overcome the above obstacles. The drug molecules can be encapsulated in the interior or adsorbed on the surface by physical action or can be connected to the framework or matrix of the nanocarrier by chemical bonding [3,4]. At present, nanocarriers have been widely used to deliver drugs [5], peptides [6], and nucleic acids [7]. Nanocarriers can (1) help drugs avoid rapid clearance during circulation and prolong their time in the blood [8], (2) be enriched at the lesion site through enhanced permeability and retention effect (EPR effect) or active targeting, which improves the utilization of drugs and reduces toxic and side effects [9], and (3) realize the controlled release of drugs through internal (e.g., pH) or external (e.g., radiation) stimulation signals [10]. Some DDSs have realized the transformation from laboratory to clinical [11].

The materials for constructing nanocarriers need to have good biocompatibility and biodegradability. A large number of organic and inorganic materials have been studied to construct carriers [12,13]. As a kind of natural polymer, polysaccharides are non-toxic, biodegradable, and rich in reserves, and have shown great application prospects in the fields of biology, medicine and pharmaceuticals [14,15,16]. Polysaccharides have various resources from terrestrial and marine animals, plants, and microorganisms. Marine polysaccharides bear important physiological functions in marine organisms and are an important type of biomass. Compared with polysaccharides extracted from the land, polysaccharides extracted from marine organisms (shells, crabs, shrimps, sharks, squids, seaweed, etc.) have absolute advantages in terms of biodiversity and simple preparation process. Marine polysaccharides and their derivatives are excellent substrates for the construction of DDSs.

Some marine polysaccharides have biological activities, such as anti-tumor, anti-viral, anti-cardiovascular disease, and immune regulatory effects [17,18]. For example, hyaluronic acid (HA) can be specifically targeted to the CD44 receptor that is overexpressed on many tumor cells [19]. Chitosan (CS) has effective antibacterial ability [20]. Besides, there are a large number of active functional groups on the backbone of marine polysaccharides, such as hydroxyl, amino, and carboxylic acid groups. Marine polysaccharides can be chemically modified through these active sites to expand their application fields [21]. Drug molecules can be conjugated to the backbone of polysaccharide molecules through cleavable chemical bonds [22]. The charged drug molecules can form nanoparticles with charged polysaccharides through electrostatic interaction [23,24]. Drug molecules can be encapsulated in the internal cavities of micelles formed by polysaccharide-based amphiphilic polymers through hydrophobic interaction [25]. The construction of the marine polysaccharide-based DDS can make DDSs possess the various advantages of nanoscale systems while also possessing the properties of polysaccharides.

This review summarizes the advantages and disadvantages of marine polysaccharides and introduces the preparation and modification methods of marine polysaccharide-based DDSs in detail. We look forward to the future applications of marine polysaccharides, and hope that this review will inspire the research and development of marine polysaccharide products.

## 2. Characteristics of Marine Polysaccharides

### 2.1. Structure and Classification

Polysaccharides are a class of carbohydrates with complex and large molecular structures. Polysaccharides are formed by the dehydration of multiple monosaccharide molecules. The structural units of polysaccharides are connected by glycoside bonds. Common glycoside bonds include α-1,4-, β-1,4- and α-1,6-glycosidic bonds. The structural unit can be connected into a straight chain or a branched chain. The straight chain is generally connected by α-1,4-glycosidic bonds (such as starch) or β-1,4-glycosidic bonds (such as cellulose); the connection point in the branched chain is often α-1,6-glycosidic bonds [26,27]. The polysaccharides composed of single monosaccharide are defined as homopolysaccharides, such as starch, cellulose, and glycogen [26]. The polysaccharides composed of diverse monosaccharides are called heteropolysaccharides such as HA, chondroitin, and alginate (Alg) [28,29,30]. Marine polysaccharides can be divided into marine animal polysaccharides, marine plant polysaccharides, and marine microbial polysaccharides, according to their sources. There are many kinds of active polysaccharides isolated from marine animals, such as chitin in crustaceans, chondroitin sulfate in cartilaginous fish bones, sulfated polysaccharides in sea cucumbers and starfish, and glycosamines in mollusks, scallops, clams, and abalones. The seaweeds (such as brown algae, red algae, and green algae) are the main source of marine plant polysaccharides. Brown algae are rich in algin and fucoidan; red algae mainly contain carrageenan and agar polysaccharides; the Ulva polysaccharide is the main ingredient in green algae. Marine microbial polysaccharides are polysaccharides produced by marine bacteria, microalgae, or fungi. The chemical structure, source, and feature of typical marine polysaccharides are summarized in Table 1.

### 2.2. Advantages of Marine Polysaccharide-Based DDSs

**Figure 1:**
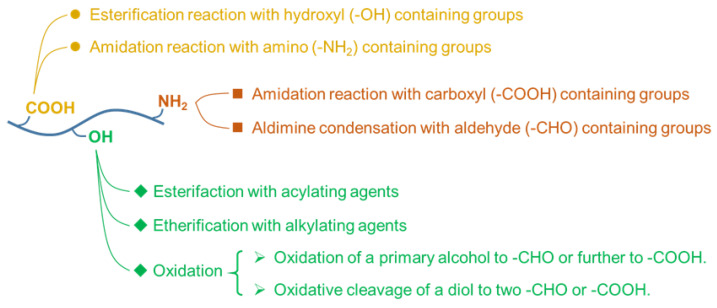
Overview of polysaccharide derivatization.

Biocompatibility, biodegradability, low immunogenicity, and high natural availability are recognized advantages of marine polysaccharides [31,32]. The presence of multifunctional groups (such as hydroxyl, carboxyl, and amine) on the molecular backbone makes it easy to be modified by chemical, biochemical, or enzymatic modification (Figure 1). Common construction methods include: (i) esterification of hydroxyl groups with acylating agents; etherification of hydroxyl groups with alkylation agents; oxidation of primary alcohols to carboxyl groups; oxidation of vicinal secondary hydroxyl groups to aldehydes; (ii) ester bonds consist of hydroxyl groups linked to carboxyl groups; amide bonds consist of carboxyl groups linked to amino groups; hydrazone bond formed by the reaction of -COOH and -NHNH_2_; (iii) interaction between amino groups and hydroxyl or carboxyl groups. Drug molecules can be grafted onto polysaccharides through the reaction with the active groups on the polysaccharides. Carboxymethyl groups can be introduced into the polysaccharide backbone through the esterification reaction of polysaccharides and carboxylic acid derivatives. Carboxymethylation can increase the solubility and electronegativity of polysaccharides. In addition, some types of marine polysaccharides have unique physicochemical properties and pharmacological effects due to their unique structure. For instance, CS is the only alkaline polysaccharide in nature. The amino groups in its molecular chain can combine with protons to generate cations in weak acid solutions, thus having a broad-spectrum antibacterial activity [33,34]. Some marine polysaccharides retain several recognition functions, permitting specific receptor recognition or adhesion, as well as providing neutral coatings with low surface energy and avoiding non-specific protein adsorption. HA can recognize the CD44 receptor on the cell surface [35]. The carrier with the negative surface can prevent the adhesion of proteins in the blood and prolong the circulation time of the carrier. Marine polysaccharides such as HA and Alg exhibit negative charge under physiological conditions, and in some cases, can be used as a substitute for poly(ethylene glycol) (PEG) segments. The mentioned advantages make marine polysaccharides an ideal candidate material for the design and preparation of DDSs.

### 2.3. Drawbacks of Using Polysaccharide in Drug Delivery

Although many marine polysaccharide-based products, such as wound dressings and dermal filler, have been used clinically, most of these products are used in vitro or in specific locations in the body [36,37]. As for the carrier, it is usually required to have a clear structure and clear metabolism in the body [38,39]. Marine polysaccharide-based materials are at a disadvantage in this regard. Because the source of marine polysaccharides is destined to have its molecular weight and structure susceptible to the season and place of production [40]. Even though high-quality and stable products can be obtained through industrial refinement, it is usually accompanied by a substantial increase in cost. In addition, due to the uncertainty of selecting model drugs in most studies, it is necessary to fully study the interaction between drug molecules, polysaccharides, and the human body, including absorption, distribution, metabolism, and excretion.

Some marine polysaccharides have poor solubility in common solvents, which limits the chemical modification of polysaccharides. For example, CS can only be dissolved in some dilute inorganic or organic acids. Only by reducing the molecular weight or making hydrophilic modification can the water solubility of CS be increased [41]. Alg is soluble in an aqueous solution, but after multiple steps of modification, its solubility in an aqueous solution is usually greatly reduced. Considering these drawbacks, the design of DDSs based on marine polysaccharides should be considered holistically.

**Figure 2:**
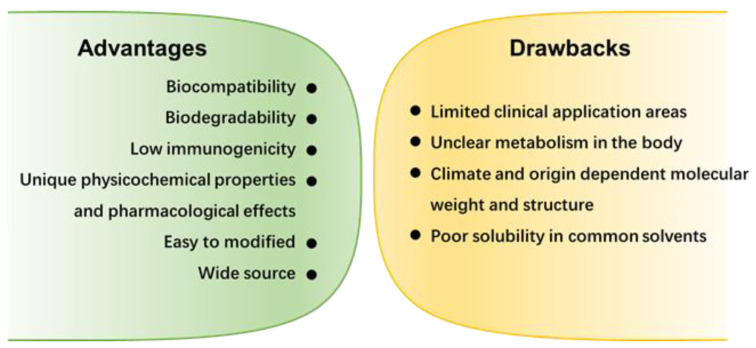
Advantages and disadvantages of marine polysaccharides in the construction of DDSs.

In fact, some of the drawbacks mentioned above are not only the existence of marine polysaccharide-based DDSs, but carriers of other materials also face these problems. Therefore, starting from the bottom, optimizing the design, flexible modification, the all-around carrier may be obtained. Figure 2 summarizes the advantages and disadvantages of marine polysaccharides in the construction of DDSs.

## 3. Preparation and Modification of the Marine Polysaccharide-Based DDS

### 3.1. Preparation of Marine Polysaccharide-Based Nanoparticles (NPs)

The preparation of marine polysaccharide-based NPs can be categorized as self-assembly and covalent crosslinking. Self-assembled NPs are formed under the action of non-covalent bonds. The driving force mainly includes polyelectrolyte complexation, hydrophobic interaction, and ionic interactions [21,42]. There are many ways to achieve covalent crosslinking, such as chemical crosslinking and radiation crosslinking [43,44]. Depending on the synthesis method, the obtained NPs can be nanogels, micelles, or vesicles [45,46,47].

#### 3.1.1. Polyelectrolyte Complexation (PEC)

**Figure 3:**
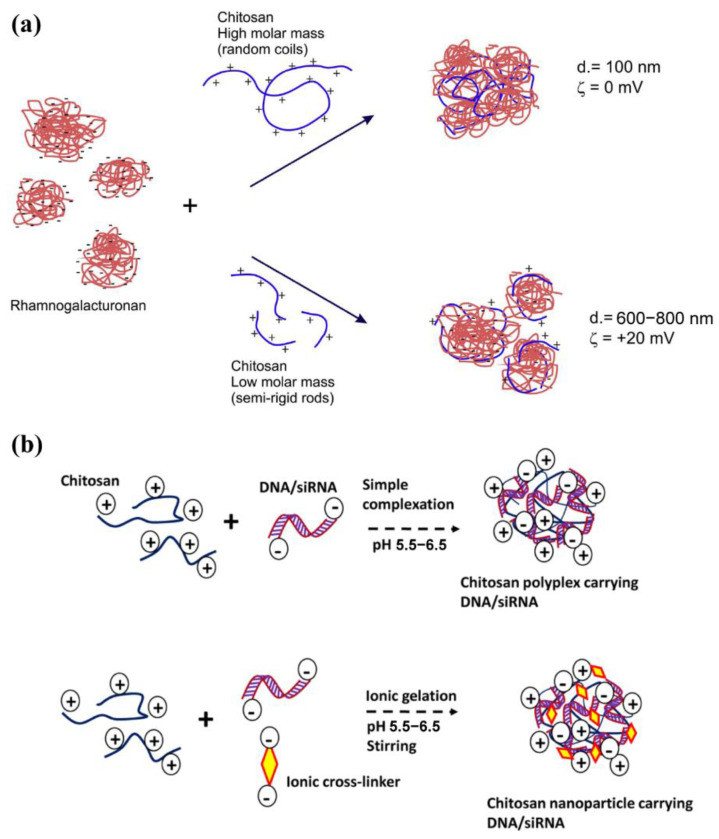
(**a**) Schematic representation of the effect of CS molar mass on the particle size of PEC formed with semi-flexible polynion, adapted from [54]. (**b**) Common preparation methods of chitosan nanocarrier for DNA/siRNA delivery. Adapted from [55].

Marine polysaccharides containing ionizable groups can be classified as natural polyelectrolytes. The polyelectrolyte complexation is formed by electrostatic interaction between oppositely charged components. Naturally charged polysaccharides can easily form PEC with oppositely charged polyelectrolytes. The only cationic polysaccharide, CS, can form PEC with negative polysaccharides, peptides, and polyacrylic acid family [48]. Furthermore, CS can also form complexes with nucleic acids, serving as a matrix for gene carriers [49]. However, it should be noted that due to the poor water solubility of CS, more studies have been conducted on CS derivatives, such as glycol chitosan [50,51]. Malhotra et al. used sodium hydride to catalyze the etherification reaction between chlorinated chitosan and methyl-PEG, and PEG-grafted chitosan was successfully synthesized [50]. Under physiological conditions, some groups become negatively charged after ionization, such as the carboxylate in HA and Alg, and the sulfonate in chondroitin sulfate, thus that polysaccharide molecules can interact with positively charged polymers [52,53]. Figure 3a shows a schematic diagram of the formation of NPs by electrostatic interaction between CS and semi-flexible polyethylene glycol [54]. The molecular weight and flexibility of each component will affect the particle size. Similarly, the NPs formed by the complexation of CS and nucleic acid are also affected by factors such as the ratio of nitrogen to phosphorus and pH (Figure 3b) [55]. At a low N/P ratio, cationic compounds are not enough to compress nucleic acids into nanoparticles, and loose complexes with low transfection and low toxicity efficiency can be formed through weak electrostatic interaction. As the N/P ratio increases, when the cationic compounds reach a sufficient amount, compact complexes with ideal transfection efficiency but quite a cytotoxicity can be formed [7]. Both the nucleic acid and the cell membrane are negatively charged, thus the charge of the carrier not only affects the loading of the nucleic acid but also affects the affinity of the nanocarrier and the cell membrane. These positively charged nanocarriers are easily attached to the cell membrane surface and then taken up by the cell. The main advantage of this method for preparing marine polysaccharide-based NPs is a simple operation. NPs can be formed in situ by simply mixing two polyelectrolytes with opposite charges in a solution. However, the stability of the complex requires attention. The formation and stability of the PEC depend on the structure, molecular weight, surface charge density, and mixing ratio of the polyelectrolyte. External conditions such as pH, temperature, ionic strength, and solvent properties also affect the preparation process of NPs.

#### 3.1.2. Hydrophobic Interaction

**Figure 4:**
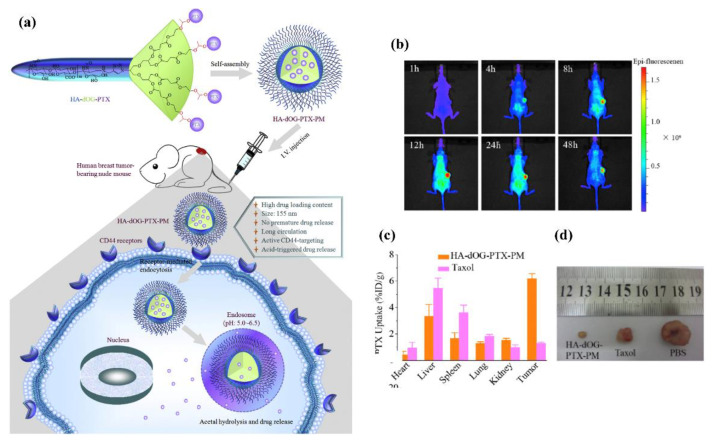
(**a**) Endosomal pH-activatable HA-bdendritic oligoglycerol (HA-dOG-PTX-PM) for active CD44-targeted paclitaxel (PTX) delivery in vivo; (**b**) in vivo fluorescence images of MCF-7 human breast tumor-bearing nude mice at different time points following injection of DIR-loaded HA-dOG-PTX-PM; (**c**) quantification of PTX accumulated in tumor and different organs using HPLC measurements. PTX uptake is expressed as injected dose per gram of tissue (%ID/g). Data are presented as mean ± SD (*n* = 3); (**d**) photographs of typical tumor blocks collected from different treatment groups of mice on day 29. Adapted from [59].

Micelle is a thermodynamically stable nanosystem self-assembled of amphiphilic polymers in an aqueous solution. The hydrophobic cavity of the micelle can be loaded with poorly water-soluble drugs to realize the solubilization of the drug; the hydrophilic shell of the micelle can protect the drug from non-specific uptake by the reticuloendothelial system and prolong the retention of the drug in the blood circulation. When hydrophilic polysaccharides are grafted with hydrophobic fragments, amphiphilic copolymers based on polysaccharides are obtained [56,57]. Commonly used hydrophobic fragments include cholesterol, steroid acids, deoxycholic acid, and hydrophobic polymers [58]. The hydroxyl, amino, and carboxyl groups on the polysaccharide backbone are common sites for connecting hydrophobic fragments. When in an aqueous solution, to achieve the minimum free energy, the hydrophobic segment can spontaneously form micelles or self-aggregates through the interaction between the intermolecular and intramolecular hydrophobic parts [58]. For instance, Zhong et al. used HA (*M*_W_ ~ 9.5 kDa) as the hydrophilic segment to prepare endosomal pH-activatable paclitaxel prodrug micelles for active targeting and effective treatment of CD44-overexpressing human breast cancer xenografts in nude mice (Figure 4) [59]. The in vivo pharmacokinetics and biodistribution studies showed that the HA-shelled acid-activatable paclitaxel prodrug micelles (HA-dOG-PTX-PM) had a prolonged circulation time in the nude mice and a remarkably high accumulation in the MCF-7 tumor (6.19%ID/g at 12 h post-injection). The size and thermodynamic stability of micelles depend on the ratio of hydrophilic and hydrophobic parts.

#### 3.1.3. Ionic Interaction

**Figure 5:**
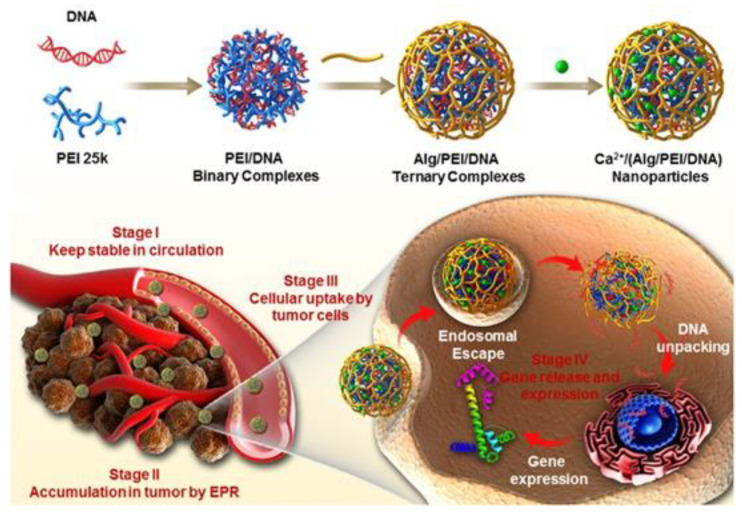
Preparation of Ca^2+^/(Alg/PEI/DNA) NPs and the schematic illustration of the in vivo transportation process of Ca^2+^/(Alg/PEI/DNA) NPs. Adapted from [62].

The ionic interaction is also a kind of electrostatic interaction. The polyelectrolyte polysaccharide interacts with oppositely charged ions to crosslink [60]. Ionic interaction strategy is the most useful method to crosslink Alg. Alg is a polysaccharide containing beta-D-mannuronate (M) and alpha-L-guluronate (G) building blocks. In the presence of divalent cations, such as Cu^2+^ or Ca^2+^, the G blocks of adjacent Alg chains could be cooperatively chelated [61]. For example, Zhang et al. prepared a gene carrier (denoted as Ca^2+^/(Alg/PEI/DNA) NPs) with calcium ions crosslinked sodium alginate as a protective layer [62]. As shown in Figure 5, sodium alginate, which was further crosslinked by Ca^2+^, was chosen as the shielding material to improve the stability of PEI/DNA complexes. Compared to PEI/DNA complexes and Alg/PEI/DNA complexes, Ca^2+^/(Alg/PEI/DNA) NPs exhibited enhanced stability, which was confirmed by the in vitro and in vivo. Furthermore, the pharmacokinetic study indicated that Ca^2+^/(Alg/PEI/DNA) NPs exhibited longer circulation time in blood, which would be beneficial to the EPR effect of NPs and could realize improved NPs accumulation at the tumor site. Factors that may affect the formation of NPs through ionic crosslinking include the molecular weight and type of polysaccharide, the ionic strength and pH of the solvent, and the ratio of ionic crosslinker to the polysaccharide [63,64]. NPs crosslinked by divalent chelation alone may not provide sufficient swelling capacity or mechanical properties because divalent ions will leak from the NPs into the surrounding medium.

#### 3.1.4. Covalent Crosslinking

The NPs after covalent crosslinking are more compact and stable. The crosslinked polysaccharide-based NPs can be used as a DDS to avoid premature dissociation and drug leakage. Usually, the reactive functional groups (such as hydroxyl, amino, and carboxylic acid groups) on the polysaccharide molecular backbone are used as crosslinking sites. The complementary group can be on the chain of another component, or an additional small molecule crosslinker can be used. Covalent crosslinking strategies mainly include Schiff-base reaction, radical polymerization, click chemistries, and photoreaction [65,66]. The vicinal glycols in some marine polysaccharides can be specifically oxidized cleavage by periodate to form aldehyde groups that could subsequently react with amine groups. The Schiff-base reaction is the most commonly used method for preparing CS-based NPs [67,68]. Molecules with more than two active groups may be used as crosslinking agents. The two aldehyde groups of glutaraldehyde can efficiently react with the amino groups on the macromolecular chain to achieve crosslinking. Schiff-base (imine) linkages would be hydrolyzed under acidic conditions, which is related to the degradation of the DDS and the controlled release of drugs. It should be noted that aldehyde groups are toxic to cells and can cause severe inflammation in the body. If crosslinking agents containing aldehyde groups are introduced during the preparation process, especially small molecule crosslinking agents (e.g., glutaraldehyde), the residual crosslinking agents should be completely removed, and the biocompatibility of the DDS needs to be strictly evaluated. Dialysis and washing against distilled water are common methods to remove residual crosslinking agents. Marine polysaccharides containing carboxyl groups (such as HA and Alg) can be crosslinked by the condensation reaction of -COOH/-OH or -COOH/-NH_2_. Ester bonds or amide bonds are formed as linkages to connect different molecular chains [28]. The polysaccharide can also be modified in advance to introduce other chemically reactive groups. Alg is usually crosslinked through ionic interaction, but the carboxyl group on its backbone can also be modified [69]. More and more new types of crosslinking agents or methods have been reported to solve the conflict between crosslinking efficiency and toxicity, such as silane coupling agents, amine-reactive disuccinimidyl tartrate, and horseradish peroxidase-catalyzed crosslinking [70,71,72].

Crosslinking methods, including physical crosslinking and chemical crosslinking methods, are the most effective methods for preparing marine polysaccharide NPs. There are other methods for preparing polysaccharide particles, such as the precipitation/coagulation method, solvent evaporation method, and spray drying method [73,74,75]. However, the particle size obtained by these methods is relatively large and uneven. The DDS prepared by these methods is suitable for mucosal absorption administration on the skin, eyes, cavities, etc., but is not suitable for intravenous administration.

### 3.2. Modification of Polysaccharide-Based NPs

A qualified DDS needs to have good dispersion stability, biocompatibility, stealth during the circulation in the body, and targeting of the lesion [76,77]. Although polysaccharide-based NPs have some inherent properties, such as good biocompatibility, to be a perfect carrier, they need to be modified to be endowed with new functions and meet the requirements of biomedical applications. Therefore, surface modification is another important step in the preparation of the polysaccharide-based DDS.

Similar to the description in the covalent crosslinking section, the modification of polysaccharide-based NPs is also based on the reactive groups in the molecule. However, compared to covalent crosslinking, the methodology used in the modification process is more flexible. Taking the functional groups on the backbone as a starting point, the polysaccharide couples to other molecules through the reaction between the functional groups [78,79]. The main methods of polysaccharide modification are the formation of esters or ethers using saccharides hydroxyl groups as nucleophiles, the chemical oxidation of primary alcohols to aldehydes or carboxylic acids, the enzymatic oxidation of primary alcohols to uronic acid, the formation of amide bonds between the saccharides carboxyl group and heteroatomic nucleophiles, as well as the nucleophilic reactions or Schiff-base reaction of the amines [21,80]. Physical adsorption is also one of the methods for surface modification, but it is usually used for the modification of charged polysaccharide (e.g., CS, HA, Alg) [81]. The modification process may be achieved through one or more steps.

#### 3.2.1. Modification of Functional Molecules on Marine Polysaccharide-Based NPs

**Figure 6:**
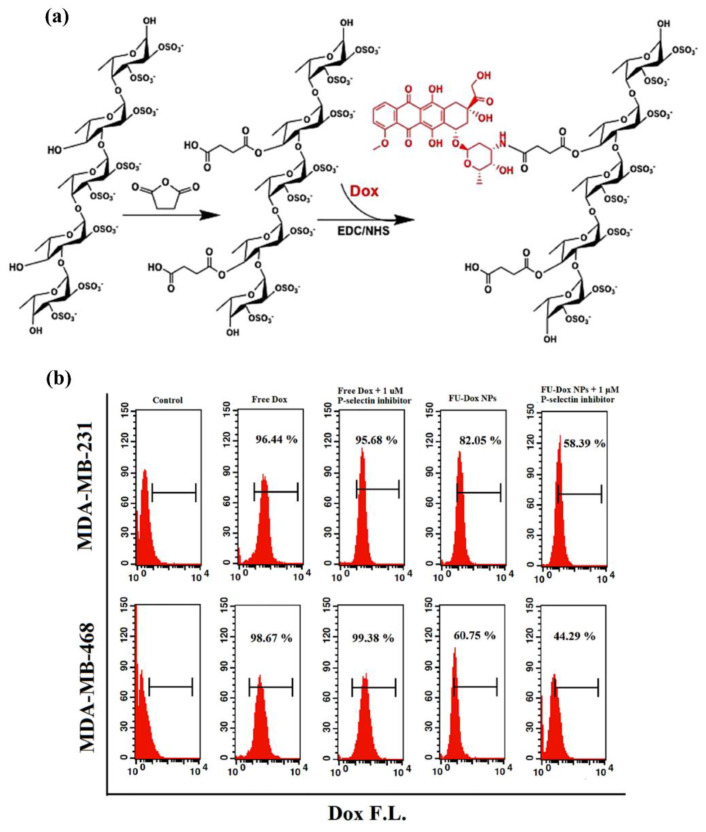
(**a**) Synthesis route of fucoidan-doxorubicin conjugate (FU-Dox NPs) developed by direct conjugation of Dox to the fucoidan backbone; (**b**) flow cytometry analysis of the cellular uptake of FU-Dox NPs after pretreatment with 1 μM P-selectin inhibitor, KF 38789, for MDA-MB-231 and MDA-MB-468 cell lines. Adapted from [84].

The purpose of the functional modification is to change the in vivo process of NPs after intravenous injection, thus that they can have long circulation or targeting functions, thereby enhancing drug efficacy and reducing adverse reactions. For marine polysaccharide-based NPs, giving DDSs the ability to target lesions actively is the main research content. Coupling or adsorbing appropriate ligands (including antibodies, haptens, lectins, folic acid, etc.) on the surface of NPs, DDSs can be directed to specific cells using the strong affinity of the ligands to specific receptors on the cell surface [82]. For example, the surface of CS-based NPs can be efficiently targeted to tumors after being conjugated with folic acid [83]. In addition, there are also reports that graft drug molecules on the polysaccharide backbone to achieve drug delivery. As illustrated in Figure 6a, Jafari et al. reported a fucoidan (*M*_W_ ~70 kDa)-based DDS for minimizing the side effects of doxorubicin (Dox) with the help of active targeting toward P-selectin [84]. P-selectin, which plays an important role in metastasis by enhancing the adhesion of cancer cells to endothelium and activated platelets in distant organs, is overexpressed on many cancer types. The fucoidan-doxorubicin conjugate (FU-Dox NPs) showed a well-controlled size distribution and sustained release. The active targeting capability of FU-Dox NPs toward P-selectin resulted in enhanced cellular uptake and cytotoxicity against the MDA-MB-231 cell line with high P-selectin expression compared to the MDA-MB-468 cell line with low P-selectin expression Figure 6b. To achieve a controlled release of the pendant, cleavable bonds will be introduced into the DDS. For example, disulfide bonds or imine bonds were introduced between the functional molecules and the polysaccharide backbone to realize the response release of the tumor microenvironment [85,86,87].

**Table 1:** The chemical structure, source, and feature of typical marine polysaccharides.

Polysaccharides	Structure	Source	Feature	Reference
Chitosan (CS)	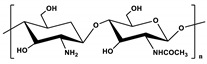	Extracted from shrimp and crab	CS is a deacetylated chitin derivative, consisting of β-1,4-linked glucosamine (2-amino-2-deoxy-β-D-glucose) and minor amounts of N-acetyl glucosamine.	[20,41]
Hyaluronic Acid (HA)	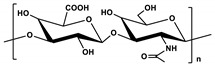	Extracted from fish eye and mussel	HA is a linear negatively charged polysaccharide constituted repeating monosaccharide unit of N-acetyl-D-glucosamine and D-glucuronic acid, which is linked together via alternating β-1,3 and β-1,4 glycosidic bonds.	[28]
Alginate (Alg)	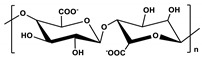	Extracted from brown algae	Alg is a well-known linear anion polyelectrolyte polysaccharide consisting of β-D-Mannuronic acid (M units) and α-L-Guluronic acid (G units).	[29,53]
Chondroitin sulfate	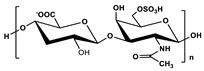	Extracted from fish cartilage	Chondroitin sulfate composed of an alternating disaccharide units of N-acetylgalactosamine and glucuronic acid, which joined together through β-(1→3) glycosidic bonds.	[30,37]
Fucoidan	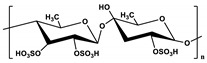	Extracted from brown algae	Fucoidan derived from kelp is formed by the sulfated fucose linked by α-(1→3) glycosidic bonds, while the fucoidan derived from fucus and ascophyllum is linked by α-(1→3) and α-(1→4) glycosidic bonds.	[68,84]
Ulva	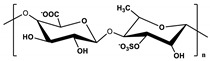	Extracted from green algae	Ulva is dominated by repeating disaccharide units, where uronic acid, either D-glucuronic acid or L-iduronic acid, or D-xylose is linked to L-rhamnose-3-sulfate through 1, 4-glycosidic bonds.	[37]
Carrageenan (λ) Carrageenan (ι) Carrageenan (K)	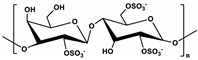 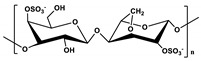 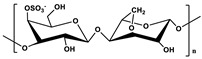	Extracted from red algae	Carrageenan is mainly formed by alternately connecting disaccharide units composed of α-(1→4)-D-galactopyranose or β-(1→3)-D-galactopyranose of substitution of sulfate groups.	[37]

#### 3.2.2. Grafting Polymer Chains onto Marine Polysaccharides

**Figure 7:**
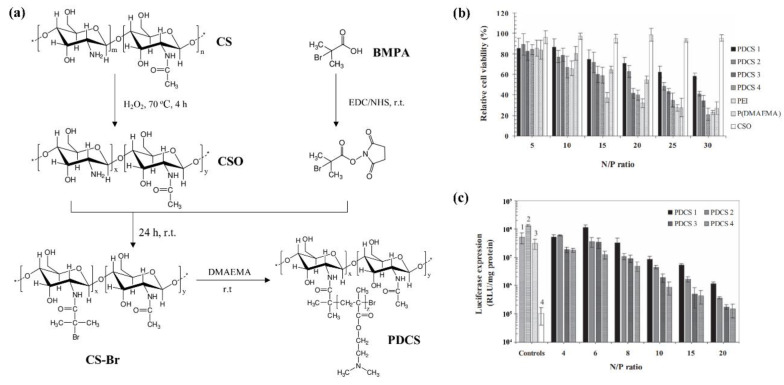
(**a**) Route of the synthesis of P(DMAEMA) functionalized CS (PDCS); (**b**) Cell viability of PDCS/pDNA polyplexes at different N/P ratios in COS7, where polyethylenimine (PEI) (25 kDa), P(DMAEMA) and chitosan oligomers (CSO) polyplexes were used as controls. (mean ± SD, *n* = 4); (**c**) in vitro gene transfection efficiency of PDCS/pDNA polyplexes in comparison with those mediated by PEI (25 kDa) (control 1) at N/P ratio of 10, ExGen 500 (control 2) at N/P ratio of 6, P(DMAEMA) (control 3) at N/P ratio of 10, and CSO (control 4) at N/P ratio of 20 in COS7 cell line in the presence of serum. (mean ± SD, *n* = 3). Adapted from [90].

Grafting polymer chains on the macromolecular backbone can effectively change the properties of marine polysaccharides. The introduction of polymer chains can be divided into two strategies: “graft from” and “graft to” [88,89]. The “graft from” strategy mainly takes the active group on the polysaccharide backbone as the initiation site and introduces functional side chains through atom transfer radical polymerization (ATRP) and reversible addition-fragmentation chain transfer polymerization (RAFT), including cationic components for nucleic acid delivery, PEGylated, and zwitterionic moieties for shielding effects, and functional species for bioimaging applications as well as bioresponsive drug release applications [15]. Ping et al. used ATRP to functionalize CS in a well-controlled manner [90]. As shown in Figure 7a, a series of new degradable cationic polymers (termed as PDCS) composed of biocompatible CS (*M*_w_ ~ 150 kDa; degree of deacetylation: 83%) backbones and poly((2-dimethyl amino)ethyl methacrylate) (P(DMAEMA)) side chains of different length were designed as highly efficient gene vectors via ATRP. In comparison with high-molecular-weight P(DMAEMA) and ‘gold-standard’ PEI (25 kDa), the PDCS vectors showed considerable buffering capacity in the pH range of 7.4 to 5. They were capable of mediating much more efficient gene transfection at low N/P ratios (Figure 7b). At their own optimal N/P ratios for transfection, the PDCS/pDNA complexes showed much lower cytotoxicity (Figure 7c). The “graft to” strategy is usually based on more reactive reactions such as click chemistry reactions [47]. The properties of DDSs can be controlled by regulation of the length of the polymer segment [15].

## 4. Conclusions and Perspectives

Marine polysaccharides are gifts from nature. The development and utilization of marine polysaccharides are one of the ways to realize the high value of marine resources. The inherent natural properties of marine polysaccharides, such as biodegradability and biocompatibility, make marine polysaccharide-based nanocarriers a high potential platform for developing DDS. The marine polysaccharide-based DDS integrates the advantages of nanotechnology and is suitable as a carrier for different pharmaceutical preparations. From the bench to industrialization is not a simple process. It is necessary to further optimize the extraction and purification process of marine polysaccharides and integrate upstream and downstream resources. With the development and mutual penetration of related disciplines such as chemistry, biology, physics, and pharmacy, the application fields of marine polysaccharide products have gradually expanded, for example, in the fields of tissue engineering, scaffold materials, and wound accessories. There are already a large number of products based on marine polysaccharides, such as clinical wound dressings (e.g., Hemcon Gauze, Regenecare HA) and health products (e.g., Move Free joint health supplements). However, the marine polysaccharide-based DDS still has a long way to go to clinical application. As technical issues such as preparation, quality standards, and route of administration are resolved, marine polysaccharide-based products will have great development prospects.

## Data Availability

Data sharing not applicable.

## Abbreviations

DDS	Drug delivery system
NDDS	Nano drug delivery system
HA	Hyaluronic acid
CS	Chitosan
CSO	Chitosan oligomers
Alg	Alginate
NPs	Nanoparticles
PEC	Polyelectrolyte complexation
PTX	Paclitaxel
EPR	Enhanced permeability and retention
Dox	Doxorubicin
ATRP	Atom transfer radical polymerization
RAFT	Reversible addition-fragmentation chain transfer polymerization
P(DMAEMA)	Poly((2-dimethyl amino)ethyl methacrylate)
PEI	Polyethylenimine

## References

[1] Sun Q.H., Zhou Z.X., Qiu N.S., Shen Y.Q. (2017). Rational design of cancer nanomedicine: Nanoproperty integration and synchronization. Adv. Mater..

[2] Cong H.L., Zhou L.P., Meng Q.Y., Zhang Y.X., Yu B., Shen Y.Q., Hu H. (2019). Preparation and evaluation of PAMAM dendrimer-based polymer gels physically cross-linked by hydrogen bonding. Biomater. Sci..

[3] Park K. (2014). Controlled drug delivery systems: Past forward and future back. J. Control. Release.

[4] Sun Y.Z., Jing X.D., Ma X.L., Feng Y.L., Hu H. (2020). Versatile types of polysaccharide-based drug delivery systems: From strategic design to cancer therapy. Int. J. Biol. Macromol..

[5] Meng Q.Y., Cong H.L., Hu H., Xu F.J. (2020). Rational design and latest advances of codelivery systems for cancer therapy. Mater. Today Bio..

[6] Kovalainen M., Monkare J., Riikonen J., Pesonen U., Vlasova M., Salonen J., Lehto V.P., Jarvinen K., Herzig K.H. (2015). Novel delivery systems for improving the clinical use of peptides. Pharmacol. Rev..

[7] Hu H., Yuan W., Liu F.S., Cheng G., Xu F.J., Ma J. (2015). Redox-responsive polycation-functionalized cotton cellulose nanocrystals for effective cancer treatment. ACS Appl. Mater. Interfaces.

[8] Sun Y., Hu H., Jing X., Meng Q.Y., Yu B., Cong H.L., Shen Y.Q. (2021). Co-delivery of chemotherapeutic drugs and cell cycle regulatory agents using nanocarriers for cancer therapy. Sci. China Mater.

[9] Kumari P., Ghosh B., Biswas S. (2016). Nanocarriers for cancer-targeted drug delivery. J. Drug Target..

[10] Sun Y., Ma X., Hu H. (2021). Application of Nano-Drug Delivery System Based on Cascade Technology in Cancer Treatment. Int. J. Mol. Sci..

[11] Bulbake U., Doppalapudi S., Kommineni N., Khan W. (2017). Liposomal formulations in clinical use: An updated review. Pharmaceutics.

[12] Liu Y.L., Chen D., Shang P., Yin D.C. (2019). A review of magnet systems for targeted drug delivery. J. Control. Release.

[13] Wang Y., Zhao Q.F., Han N., Bai L., Li J., Liu J., Che E.X., Hu L., Zhang Q., Jiang T.Y. (2015). Mesoporous silica nanoparticles in drug delivery and biomedical applications. Nanomedicine.

[14] Meng Q.Y., Sun Y., Cong H.L., Hu H., Xu F.J. (2021). An overview of chitosan and its application in infectious diseases. Drug Deliv. Transl. Res..

[15] Hu Y., Li Y., Xu F.J. (2017). Versatile functionalization of polysaccharides via polymer grafts: From design to biomedical applications. Acc. Chem. Res..

[16] Guo X., Wang Y., Qin Y.M., Shen P.L., Peng Q. (2020). Structures, properties and application of alginic acid: A review. Int. J. Biol. Macromol..

[17] Manivasagan P., Oh J. (2016). Marine polysaccharide-based nanomaterials as a novel source of nanobiotechnological applications. Int. J. Biol. Macromol..

[18] Cardoso M.J., Costa R.R., Mano J.F. (2016). Marine origin polysaccharides in drug delivery systems. Mar. Drugs.

[19] Oh E.J., Park K., Kim K.S., Kim J., Yang J.A., Kong J.H., Lee M.Y., Hoffman A.S., Hahn S.K. (2010). Target specific and long-acting delivery of protein, peptide, and nucleotide therapeutics using hyaluronic acid derivatives. J. Control. Release.

[20] Moeini A., Pedram P., Makvandi P., Malinconico M., d’Ayala G.G. (2020). Wound healing and antimicrobial effect of active secondary metabolites in chitosan-based wound dressings: A review. Carbohydr. Polym..

[21] Debele T.A., Mekuria S.L., Tsai H.C. (2016). Polysaccharide based nanogels in the drug delivery system: Application as the carrier of pharmaceutical agents. Mater. Sci. Eng. C.

[22] Meng Q.Y., Hu H., Zhou L.P., Zhang Y.X., Yu B., Shen Y.Q., Cong H.L. (2019). Logical design and application of prodrug platforms. Polym. Chem..

[23] Nitta S.K., Numata K. (2013). Biopolymer-Based Nanoparticles for Drug/Gene Delivery and Tissue Engineering. Int. J. Mol. Sci..

[24] Huh M.S., Lee E.J., Koo H., Yhee J.Y., Oh K.S., Son S., Lee S., Kim S.H., Kwon L.C., Kim K. (2017). Polysaccharide-based Nanoparticles for Gene Delivery. Top. Curr. Chem..

[25] Liu Y., Sun J., Zhang P., He Z. (2011). Amphiphilic polysaccharide-hydrophobicized graft polymeric micelles for drug delivery nanosystems. Curr. Med. Chem..

[26] Habibi Y., Lucia L.A., Rojas O.J. (2010). Cellulose nanocrystals: Chemistry, self-assembly, and applications. Chem. Rev..

[27] Perez S., Bertoft E. (2010). The molecular structures of starch components and their contribution to the architecture of starch granules: A comprehensive review. Starch/Staerke.

[28] Collins M.N., Birkinshaw C. (2013). Hyaluronic acid based scaffolds for tissue engineering-A review. Carbohydr. Polym..

[29] Lee K.Y., Mooney D.J. (2012). Alginate: Properties and biomedical applications. Prog. Polym. Sci..

[30] Muzzarelli R.A.A., Greco F., Busilacchi A., Sollazzo V., Gigante A. (2012). Chitosan, hyaluronan and chondroitin sulfate in tissue engineering for cartilage regeneration: A review. Carbohydr. Polym..

[31] Yu Y., Shen M.Y., Song Q.Q., Xie J.H. (2018). Biological activities and pharmaceutical applications of polysaccharide from natural resources: A review. Carbohydr. Polym..

[32] Hu H., Xu F.J. (2020). Rational design and latest advances of polysaccharide-based hydrogels for wound healing. Biomater. Sci..

[33] Rabea E.I., Badawy M.E.T., Stevens C.V., Smagghe G., Steurbaut W. (2003). Chitosan as antimicrobial agent: Applications and mode of action. Biomacromolecules.

[34] Dash M., Chiellini F., Ottenbrite R.M., Chiellini E. (2011). Chitosan-A versatile semi-synthetic polymer in biomedical applications. Prog. Polym. Sci..

[35] Lesley J., Hyman R., English N., Catterall J.B., Turner G.A. (1997). CD44 in inflammation and metastasis. Glycoconj. J..

[36] Gupta A., Kowalczuk M., Heaselgrave W., Britland S.T., Martin C., Radecka I. (2019). The production and application of hydrogels for wound management: A review. Eur. Polym. J..

[37] Jing X., Sun Y.D., Ma X., Hu H. (2021). Marine polysaccharides: Green and recyclable resources as wound dressings. Mater. Chem. Front..

[38] Elsabahy M., Wooley K.L. (2012). Design of polymeric nanoparticles for biomedical delivery applications. Chem. Soc. Rev..

[39] Haag R., Kratz F. (2006). Polymer therapeutics: Concepts and applications. Angew. Chem. Int. Ed..

[40] Garcia-Gonzalez C.A., Alnaief M., Smirnova I. (2011). Polysaccharide-based aerogels-promising biodegradable carriers for drug delivery systems. Carbohydr. Polym..

[41] Pillai C.K.S., Paul W., Sharma C.P. (2009). Chitin and chitosan polymers: Chemistry, solubility and fiber formation. Prog. Polym. Sci..

[42] Liu M., Li H., Wang X., Jing L., Jiang P., Li Y. (2020). Experimental study of the vascular normalization window for tumors treated with apatinib and the efficacy of sequential chemotherapy with apatinib in lung cancer-bearing mice and patients. Cancer Med..

[43] Li M.Q., Tang Z.H., Zhang D.W., Sun H., Liu H.Y., Zhang Y., Zhang Y.Y., Chen X.S. (2015). Doxorubicin-loaded polysaccharide nanoparticles suppress the growth of murine colorectal carcinoma and inhibit the metastasis of murine mammary carcinoma in rodent models. Biomaterials.

[44] Zhao L., Mitomo H. (2009). Hydrogels of dihydroxypropyl chitosan crosslinked with irradiation at paste-like condition. Carbohydr. Polym..

[45] Soni K.S., Desale S.S., Bronich T.K. (2016). Nanogels: An overview of properties, biomedical applications and obstacles to clinical translation. J. Control. Release.

[46] Zhang N., Wardwell P.R., Bader R.A. (2013). Polysaccharide-based micelles for drug delivery. Pharmaceutics.

[47] Schatz C., Louguet S., Le Meins J.F., Lecommandoux S. (2009). Polysaccharide-block-polypeptide copolymer vesicles: Towards synthetic viral capsids. Angew. Chem. Int. Ed..

[48] Hamman J.H. (2010). Chitosan based polyelectrolyte complexes as potential carrier materials in drug delivery systems. Mar. Drugs.

[49] Mao S.R., Sun W., Kissel T. (2010). Chitosan-based formulations for delivery of DNA and siRNA. Adv. Drug Deliv. Rev..

[50] Malhotra M., Lane C., Tomaro-Duchesneau C., Shyamali S., Prakash S. (2011). A novel method for synthesizing PEGylated chitosan nanoparticles: Strategy, preparation, and in vitro analysis. Int. J. Nanomed..

[51] Chen S., Deng J., Zhang L.M. (2021). Cationic nanoparticles self-assembled from amphiphilic chitosan derivatives containing poly(amidoamine) dendrons and deoxycholic acid as a vector for co-delivery of doxorubicin and gene. Carbohydr. Polym..

[52] Yin X., Xie H.G., Li R.X., Yan S.G., Yin H. (2021). Regulating association strength between quaternary ammonium chitosan and sodium alginate via hydration. Carbohydr. Polym..

[53] Mirtic J., Ilas J., Kristl J. (2018). Influence of different classes of crosslinkers on alginate polyelectrolyte nanoparticle formation, thermodynamics and characteristics. Carbohydr. Polym..

[54] Magalhaes G.A., Neto E.M., Sombra V.G., Richter A.R., Abreu C., Feitosa J.P.A., Paula H.C.B., Goycoole F.M., de Paula R.C.M. (2016). Chitosan/Sterculia striata polysaccharides nanocomplex as a potential chloroquine drug release device. Int. J. Biol. Macromol..

[55] Babu A., Ramesh R. (2017). Multifaceted applications of chitosan in cancer drug delivery and therapy. Mar. Drugs.

[56] Wu J.L., Tian G.X., Yu W.J., Jia G.T., Sun T.Y., Gao Z.Q. (2016). pH-Responsive hyaluronic acid-based mixed micelles for the hepatoma-targeting delivery of doxorubicin. Int. J. Mol. Sci..

[57] Shen Y., Li Q., Tu J.S., Zhu J.B. (2009). Synthesis and characterization of low molecular weight hyaluronic acid-based cationic micelles for efficient siRNA delivery. Carbohydr. Polym..

[58] Mizrahy S., Peer D. (2012). Polysaccharides as building blocks for nanotherapeutics. Chem. Soc. Rev..

[59] Zhong Y.N., Goltsche K., Cheng L., Xie F., Meng F.H., Deng C., Zhong Z.Y., Haag R. (2016). Hyaluronic acid-shelled acid-activatable paclitaxel prodrug micelles effectively target and treat CD44-overexpressing human breast tumor xenografts in vivo. Biomaterials.

[60] Zhang Y., Cai L.L., Li D., Lao Y.H., Liu D.Z., Li M.Q., Ding J.X., Chen X.S. (2018). Tumor microenvironment-responsive hyaluronate-calcium carbonate hybrid nanoparticle enables effective chemotherapy for primary and advanced osteosarcomas. Nano Res..

[61] Akay S., Heils R., Trieu H.K., Smirnova I., Yesil-Celiktas O. (2017). An injectable alginate-based hydrogel for microfluidic applications. Carbohydr. Polym..

[62] Zhang Y., Lin L., Liu L., Liu F., Maruyama A., Tian H.Y., Chen X.S. (2018). Ionic-crosslinked polysaccharide/PEI/DNA nanoparticles for stabilized gene delivery. Carbohydr. Polym..

[63] Gan Q., Wang T., Cochrane C., McCarron P. (2005). Modulation of surface charge, particle size and morphological properties of chitosan-TPP nanoparticles intended for gene delivery. Colloids Surf. B.

[64] Ma Z.S., Yeoh H.H., Lim L.Y. (2002). Formulation pH modulates the interaction of insulin with chitosan nanoparticles. J. Pharm. Sci..

[65] Zhang X., Malhotra S., Molina M., Haag R. (2015). Micro- and nanogels with labile crosslinks-from synthesis to biomedical applications. Chem. Soc. Rev..

[66] Liu Z.H., Jiao Y.P., Wang Y.F., Zhou C.R., Zhang Z.Y. (2008). Polysaccharides-based nanoparticles as drug delivery systems. Adv. Drug Deliv. Rev..

[67] Zhao W.F., Huang X.L., Wang Y.L., Sun S.D., Zhao C.S. (2016). A recyclable and regenerable magnetic chitosan absorbent for dye uptake. Carbohydr. Polym..

[68] da Silva L., Todaro V., do Carmo F.A., Frattani F.S., de Sousa V.P., Rodrigues C.R., Sathler P.C., Cabral L.M. (2018). A promising oral fucoidan-based antithrombotic nanosystem: Development, activity and safety. Nanotechnology.

[69] Lee C., Shin J., Lee J.S., Byun E., Ryu J.H., Um S.H., Kim D.I., Lee H., Cho S.W. (2013). Bioinspired, Calcium-free alginate hydrogels with tunable physical and mechanical properties and improved biocompatibility. Biomacromolecules.

[70] Nguyen M.H., Tran T.T., Hadinoto K. (2016). Controlling the burst release of amorphous drug-polysaccharide nanoparticle complex via crosslinking of the polysaccharide chains. Eur. J. Pharm. Biopharm..

[71] Mocanu G., Mihai D., Legros M., Picton L., LeCerf D. (2008). New polysaccharide-based microparticles crosslinked with siloxane: Interactions with biologically active substances. J. Bioact. Compat. Polym..

[72] Khanmohammadi M., Sakai S., Taya M. (2019). Characterization of encapsulated cells within hyaluronic acid and alginate microcapsules produced via horseradish peroxidase-catalyzed crosslinking. J. Biomater. Sci. Polym. Ed..

[73] Calvo P., RemunanLopez C., VilaJato J.L., Alonso M.J. (1997). Novel hydrophilic chitosan-polyethylene oxide nanoparticles as protein carriers. J. Appl. Polym. Sci..

[74] Park S., Hwang S., Lee J. (2011). pH-responsive hydrogels from moldable composite microparticles prepared by coaxial electro-spray drying. Chem. Eng. J..

[75] Imam M.E., Bernkop-Schnurch A. (2005). Controlled drug delivery systems based on thiolated chitosan microspheres. Drug Dev. Ind. Pharm..

[76] Zahin N., Anwar R., Tewari D., Kabir M.T., Sajid A., Mathew B., Uddin M.S., Aleya L., Abdel-Daim M.M. (2020). Nanoparticles and its biomedical applications in health and diseases: Special focus on drug delivery. Environ. Sci. Pollut. Res..

[77] Molavi F., Barzegar-Jalali M., Hamishehkar H. (2020). Polyester based polymeric nano and microparticles for pharmaceutical purposes: A review on formulation approaches. J. Control. Release.

[78] Li D., Peng X.R., Chen L., Ding J.X., Chen X.S. (2018). One-step synthesis of targeted acid-labile polysaccharide prodrug for efficiently intracellular drug delivery. ACS Biomater. Sci. Eng..

[79] Pilipenko I.M., Korzhikov-Vlakh V.A., Zakharova N.V., Urtti A., Tennikova T.B. (2020). Thermo- and pH-sensitive glycosaminoglycans derivatives obtained by controlled grafting of poly(N-isopropylacrylamide). Carbohydr. Polym..

[80] Li S., Xiong Q., Lai X., Li X., Wan M., Zhang J., Yan Y., Cao M., Lu L., Guan J. (2015). Molecular modification of polysaccharides and resulting bioactivities. Compr. Rev. Food Sci. Food Saf..

[81] Correa S., Boehnke N., Barberio A.E., Deiss-Yehiely E., Shi A., Oberlton B., Smith S.G., Zervantonakis I., Dreaden E.C., Hammond P.T. (2020). Tuning nanoparticle interactions with ovarian cancer through layer-by-layer modification of surface chemistry. Acs Nano.

[82] Muhamad N., Plengsuriyakarn T., Na-Bangchang K. (2018). Application of active targeting nanoparticle delivery system for chemotherapeutic drugs and traditional/herbal medicines in cancer therapy: A systematic review. Int. J. Nanomed..

[83] Fathi M., Zangabad P.S., Aghanejad A., Barar J., Erfan-Niya H., Omidi Y. (2017). Folate-conjugated thermosensitive O-maleoyl modified chitosan micellar nanoparticles for targeted delivery of erlotinib. Carbohydr. Polym..

[84] Jafari M., Sriram V., Xu Z.Y., Harris G.M., Lee J.Y. (2020). Fucoidan-doxorubicin nanoparticles targeting p-selectin for effective breast cancer therapy. Carbohydr. Polym..

[85] Cao J., Zheng H.R., Hu R., Liao J.H., Fei Z.M., Wei X., Xiong X., Zhang F.L., Zheng H., Li D. (2017). pH-Responsive nanoparticles based on covalently grafted conjugates of carboxymethyl chitosan and daunorubicin for the delivery of anti-cancer drugs. J. Biomed. Nanotechnol..

[86] Curcio M., Diaz-Gomez L., Cirillo G., Nicoletta F.P., Leggio A., Iemma F. (2021). Dual-targeted hyaluronic acid/albumin micelle-like nanoparticles for the vectorization of doxorubicin. Pharmaceutics.

[87] Lee S.J., Jeong Y.I. (2018). Hybrid nanoparticles based on chlorin e6-conjugated hyaluronic acid/poly(L-histidine) copolymer for theranostic application to tumors. J. Mater. Chem. B.

[88] Tizzotti M., Charlot A., Fleury E., Stenzel M., Bernard J. (2010). Modification of polysaccharides through controlled/living radical polymerization grafting-towards the generation of high performance hybrids. Macromol. Rapid Commun..

[89] Singh V., Kumar P., Sanghi R. (2012). Use of microwave irradiation in the grafting modification of the polysaccharides-A review. Prog. Polym. Sci..

[90] Ping Y., Liu C.D., Tang G.P., Li J.S., Li J., Yang W.T., Xu F.J. (2010). Functionalization of chitosan via atom transfer radical polymerization for gene delivery. Adv. Funct. Mater..

